# Monthly Record of Deaths in the City of Buffalo

**Published:** 1857-08

**Authors:** C. L. Dayton

**Affiliations:** Health Physician


ART. VIII.— *Report of Mortality in Buffalo for the month of June*, 1857. By C. L. DAYTON, M. D., Health Physician.

| DISEASES. | No. | Males. | Females. |
|---|---|---|---|
| Accidental, | 2 | 1 | 1 |
| " Fracture of the Leg, | 1 | 1 | |
| " Asphyxia Immersorum, | 5 | 4 | 1 |
| Apoplexia, | 1 | 1 | |
| Bronchitis, | 2 | | 2 |
| Croup, | 2 | 1 | 1 |
| Cholera Infantum, | 6 | 2 | 4 |
| Chloroform, Effects of Inhalation, | 1 | | 1 |
| Diarrhœa, | 1 | | 1 |
| Debilitas Senilis, | 2 | 1 | 1 |
| Dentitio, | 1 | 1 | |
| Eclampsia, | 13 | 5 | 8 |
| Febris Typhoid, | 4 | 3 | 1 |
| " Scarlatina et sequelæ, | 22 | 9 | 13 |
| " Ignotus, | 1 | 1 | |
| Gastro Enteritis, | 1 | 1 | |
| Hydrops, | 5 | 1 | 4 |
| " Cephalic, | 2 | | 2 |
| " Thoracic, | 1 | 1 | |
| Hepatitis, | 1 | 1 | |
| Hyperæmia Cerebralis, | 2 | 1 | 1 |
| " Intestinalis, | 1 | 1 | |
| Intemperance, | 4 | 2 | 2 |
| Ignotus, | 22 | 14 | 8 |
| Morbus Cordis, | 5 | 3 | 2 |
| " Coxalgium, | 1 | 1 | |
| Meningitis, | 2 | 2 | |
| Paralysis, | 1 | 1 | |
| Pertussis, | 1 | 1 | |
| Pneumonitis, | 3 | 2 | 1 |
| Rheumatism, | 2 | 2 | |
| Still-born, | 8 | 7 | 1 |
| Suicide, | 1 | | 1 |
| Tuberculosis Pulmonalis, | 12 | 8 | 4 |
| Ulceratio Intestinalis, | 1 | 1 | |
| Total, | 140 | | |

SEXES.

| | |
|---|---|
| Males, | 80 |
| Females, | 60 |
| Sex not given, | 0 |
| Total, | 140 |

AGES.

| | | | |
|---|---|---|---|
| Still-born, | 8 | Between 20 years and 30 years, | 11 |
| 1 day, | 4 | " 30 " " 40 " | 11 |
| 1 day and 30 days, | 7 | " 40 " " 50 " | 7 |
| Between 1 month and 6 months, | 13 | " 50 " " 60 " | 3 |
| " 6 months and 12 " | 15 | " 60 " " 70 " | 7 |
| " 1 year " 3 years, | 25 | " 70 " " 80 " | 1 |
| " 3 " " 5 " | 9 | " 80 " " 90 " | 0 |
| " 5 " " 10 " | 5 | " 90 " " 100 " | 0 |
| " 10 " " 20 " | 12 | " 100 " | 0 |
| Total number under 10 years, | 86 | Total number over 10 years, | 52 |

Ages not given, 2 Total, 140

NATIVITIES.

| | | | |
|---|---|---|---|
| American, (white,) | 86 | Norwegian, | 2 |
| " (colored,) | 1 | Welsh, | 0 |
| German, | 19 | French, | 0 |
| Irish, | 18 | Scotch, | 0 |
| English, | 5 | Holland, | 0 |
| Canadian, | 0 | Country not given, | 9 |

Total, 140

---

*Facial and Dental Neuralgias.*—Doctor Michel Andre recommends the following mixture for prompt relief: Extracts of opium, of belladonna, and of stramonium each 1 part; laurel water 12 parts. A few drops are placed in the meatus auditorius, and cotton is placed in the passage, taking the precaution to hold the head on the opposite side for a few moments, that the fluid may pass freely into the canal.—*Revue Therapeutique* from *Philadelphia Med. & Surg. Jour.*

---

*Ointment* for Zona, by Dr. Dutkil. Neapolitan ointment 30 grammes; ext. belladonna 4 grammes. Mix. The cure will be obtained in forty-eight hours.—*Ib.* (We have found the tincture of iodine very successful in one very bad case of the above disease.)

